# Dietary Beta-Hydroxy Beta-Methyl Butyrate Supplementation Alleviates Liver Injury in Lipopolysaccharide-Challenged Piglets

**DOI:** 10.1155/2021/5546843

**Published:** 2021-04-01

**Authors:** Yehui Duan, Bo Song, Changbing Zheng, Yinzhao Zhong, Qiuping Guo, Jie Zheng, Yulong Yin, Jianjun Li, Fengna Li

**Affiliations:** ^1^CAS Key Laboratory of Agro-ecological Processes in Subtropical Region, Hunan Provincial Key Laboratory of Animal Nutritional Physiology and Metabolic Process, National Engineering Laboratory for Pollution Control and Waste Utilization in Livestock and Poultry Production, Institute of Subtropical Agriculture, Chinese Academy of Sciences, Changsha 410125, China; ^2^University of Chinese Academy of Sciences, Beijing 100039, China; ^3^Animal Nutritional Genome and Germplasm Innovation Research Center, College of Animal Science and Technology, Hunan Agricultural University, Changsha, Hunan 410128, China

## Abstract

The current study was performed to investigate whether dietary *β*-hydroxy-*β*-methylbutyrate (HMB) could regulate liver injury in a lipopolysaccharide- (LPS-) challenged piglet model and to determine the mechanisms involved. Thirty piglets (21 ± 2 days old, 5.86 ± 0.18 kg body weight) were randomly divided into the control (a basal diet, saline injection), LPS (a basal diet), or LPS+HMB (a basal diet + 0.60% HMB-Ca) group. After 15 d of treatment with LPS and/or HMB, blood and liver samples were obtained. The results showed that in LPS-injected piglets, HMB supplementation ameliorated liver histomorphological abnormalities induced by LPS challenge. Compared to the control group, the activities of serum aspartate aminotransferase and alkaline phosphatase were increased in the LPS-injected piglets (*P* < 0.05). The LPS challenge also downregulated the mRNA expression of L-PFK, ACO, L-CPT-1, ICDH *β*, and AMPK*α*1/2 and upregulated the mRNA expression of PCNA, caspase 3, TNF-*α*, TLR4, MyD88, NOD1, and NF-*κ*B p65 (*P* < 0.05). However, these adverse effects of the LPS challenge were reversed by HMB supplementation (*P* < 0.05). These results indicate that HMB may exert protective effects against LPS-induced liver injury, and the underlying mechanisms might involve the improvement of hepatic energy metabolism via regulating AMPK signaling pathway and the reduction of liver inflammation via modulating TLR4 and NOD signaling pathways.

## 1. Introduction

As a key metabolic organ and a major site for detoxification in the body, the liver contributes importantly to protect body from bacteria and their toxic products, such as lipopolysaccharide (LPS) [1, 2]. Consequently, it is of major health and scientific significance for animals and humans to maintain the liver health. LPS is a component of the outer membrane of Gram-negative bacteria. Through the recognition by Toll-like receptor 4 (TLR4), LPS can excessively activate immune cells such as Kupffer cells (macrophages) and neutrophils [3]. The TLR4/myeloid differentiation factor 88- (MyD88-) mediated pathway can activate nuclear factor-*κ*B (NF-*κ*B) and induce productions of proinflammatory cytokines, such as tumor necrosis factor-*α* [4]. Subsequently, excessive liver inflammatory response leads to severe liver injury, such as cytoplasm vacuolization, hepatocyte karyolysis, disruption of hepatic cell cords, and inflammatory cell infiltration [5]. Moreover, inflammatory liver injury is often accompanied by insufficient energy supply in the liver [6, 7]. Therefore, modulating hepatic inflammatory response plays an essential role in attenuating LPS-induced liver injury. With the joint efforts of many researchers, several nutritional supplements (such as aspartate, N-acetylcysteine, chondroitin sulfate-rich extract of skate cartilage, and *α*-ketoglutarate) have been demonstrated to have the potential to alleviate LPS challenge-induced liver damage [6–9]. In spite of these interesting observations, it is still urgent to find low-cost drugs that doctors and animal producers are more preferable for.

*β*-Hydroxy-*β*-methylbutyrate (HMB) is a derivative of leucine and metabolized in the liver from the keto acid of leucine by *α*-ketoisocaproate dioxygenase [10]. Studies have shown that HMB is used as a nutritional supplement to exert positive roles in animals and humans under stressful or inflammatory conditions [11–13]. We recently reported that dietary supplementation with 0.60% HMB prevents muscle protein degradation and alleviates intestinal injury in LPS-challenged piglets [12, 13]. Given its beneficial roles under inflammatory condition, we hypothesized that HMB may also exert positive roles in the liver of LPS-challenged piglets. Intriguingly, recent evidence points to a strong relationship between HMB supplementation and liver health. First, HMB supplementation in patients after liver transplant has been reported to be safe and well tolerated [14]. Further evidence comes from the finding that HMB exerts beneficial effects in ameliorating insulin resistance via inhibiting glucose transporter type 2 in rat liver [15]. These metabolic enhancements that have been related to HMB make it a prime substrate to be used in subjects suffering from liver injury. Surprisingly, although most of the endogenous HMB is generated in the liver, there are few reports concerning the effects of HMB supplementation in subjects with liver disease. Therefore, further investigation is certainly necessary.

Therefore, we here seek to determine whether HMB could attenuate liver injury in LPS-challenged weanling piglets and, if so, to elucidate the underlying mechanisms. Pigs are similar to human in physiology and anatomy, and the swine model is hence considered to be a good animal model for investigating human nutrition and physiology [16, 17]. The present results will offer insight into the mechanisms of HMB's actions in the liver of piglets and also provide useful information for nutritionally ameliorating liver injury in inflammatory condition in humans.

## 2. Materials and Methods

### 2.1. Animals and Experimental Diets

The animal use protocol for this study was approved by the Committee on Animal Care of the Institute of Subtropical Agriculture, Chinese Academy of Sciences. Thirty healthy pigs (Landrace, 21 ± 2 d, barrow, 5.86 ± 0.18 kg) were chosen and randomly assigned to three groups (*n* = 10): (1) nonchallenged control (CON); (2) LPS-challenged control (LPS, *E. coli* serotype 055:B5; Sigma Chemical, St. Louis, MO, USA); (3) LPS+0.60% HMB-Ca treatment (LPS+HMB; HMB-Ca, purity = 99.2%, Ca = 13.6%, Sipu Biochemical Co. LTD, Zhangjiagang, China). The HMB-Ca and LPS doses were used in accordance with our previous studies [12]. On days 1, 3, 5, 7, 9, 11, 13, and 15 of the trial, overnight fasted piglets of the LPS and LPS+HMB groups were intraperitoneally administered LPS, whereas piglets in the CON group were injected with the same volume of sterile saline as previously described [12]. Piglets were raised individually in cages (1.80 × 1.10 m pen) and had *ad libitum* access to diets and clean drinking water. Diets were formulated to meet the nutritional needs for piglets according to the National Research Council (NRC, Supplementary Table 1) [18]. The experiment lasted for 15 days.

### 2.2. Sample Collection

All pigs were slaughtered by electrically stunning (250 V, 0.5 A, for 5-6 s) and exsanguinating at 3 h after LPS or saline injection on day 15 of the trial. Before slaughter, blood samples were obtained from the jugular vein and centrifuged at 3,000 × g at 4°C for 15 min to recover the serum, which was stored at -80°C until further analysis. After slaughter, liver samples were immediately and rapidly obtained and fixed in 4% formaldehyde or stored at -80°C until further analysis.

### 2.3. Serum Biochemical Parameters

The concentrations of serum aspartate aminotransferase (ASAT), alanine aminotransferase (ALAT), alkaline phosphatase (AKP), and glutamyl transpeptidase (GGT) were analyzed using the Biochemical Analytical Instrument (Beckman CX4) and commercial kits (Sino-German Beijing Leadman Biotech Ltd., Beijing, China).

### 2.4. Liver Morphology

After a 24 h fixation, liver morphology was examined as previously described [19].

### 2.5. Reverse Transcription and Real-Time Quantitative PCR

The quantitative RT-PCR analysis was conducted according to our previous studies [20, 21]. Briefly, total RNA of the liver tissue was extracted using Trizol reagent (Invitrogen, Carlsbad, CA, USA). The primer sequences for the selected genes were shown in Table 1. The expression of the target genes relative to housekeeping gene (*β*-actin) was determined by the 2^−△△Ct^ method [20].

### 2.6. Western Blot Analysis

Relative protein levels for claudin-1 (Invitrogen Technology, Danvers, MA, USA) and p-AMPK*α* (Thr172, Cell Signaling Technology Inc., Danvers, MA, USA) obtained from the liver tissue were determined by the Western blotting technique according to our previous studies [22–24]. The bands of the protein were visualized using a chemiluminescent reagent (Pierce, Rockford, USA) with a ChemiDoc XRS system (Bio-Rad, Philadelphia, PA, USA). We quantified the resultant signals using Alpha Imager 2200 software (Alpha Innotech Corporation, CA, USA) and normalized the data with the value of the inner control *β*-actin or the corresponding total protein.

### 2.7. Statistical Analyses

All data in this study was analyzed by the one-way ANOVA of SAS software version 9.2 (SAS Institute Inc., Cary, NC, United States), followed by a Duncan's multiple-range test to determine treatment effects. Results are presented as means with standard errors. Differences between significant means were considered as statistically different at *P* < 0.05.

## 3. Results

### 3.1. Liver Morphology

**Figure 1:**
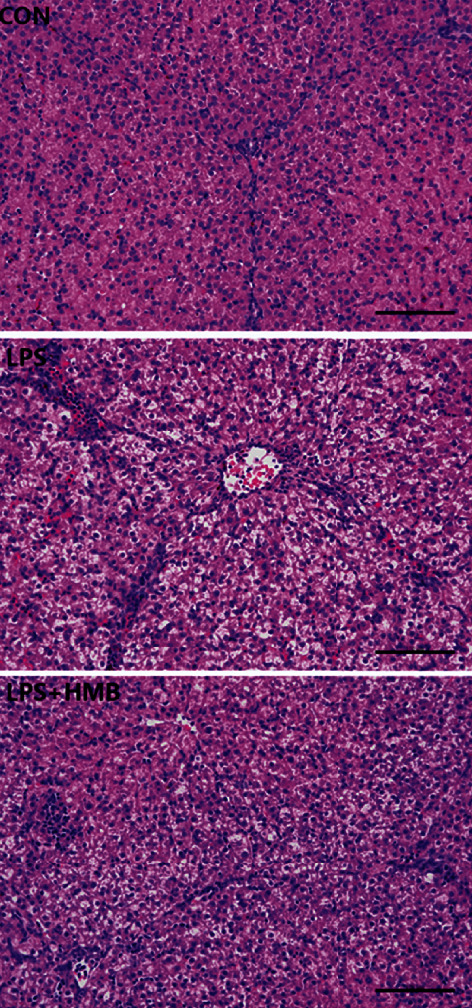
Histological examination of the liver samples of piglets injected with lipopolysaccharide (LPS) or saline. Sections were obtained with haematoxylin and eosin (×200). CON: control; HMB: *β*-hydroxy-*β*-methylbutyrate.

Compared to the CON, the pigs treated with LPS exhibited liver injury, as evidenced by hepatocyte caryolysis, karyopycnosis, hypatocyte vacuolization, and hepatic cell cords arrangement in disorder, inflammatory cell infiltration, fibroblast proliferation, and hyperaemia in hepatic sinusoids (Figures 1(a) and 1(b)). Furthermore, the impairment of liver induced by LPS was largely recovered by HMB supplementation (Figure 1(c)).

### 3.2. Serum Biochemical Parameters

**Figure 2:**
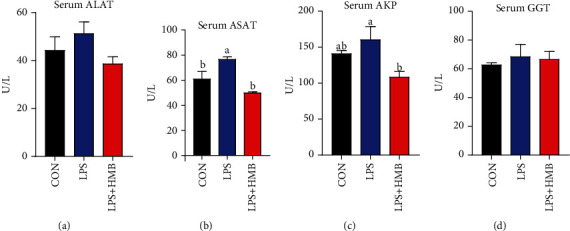
Effects of dietary supplementation of HMB on serum biochemical parameters of piglets injected with LPS or saline. (a) Aspartate aminotransferase (ASAT); (b) alanine aminotransferase (ALAT); (c) alkaline phosphatase (AKP); (d) glutamyl transpeptidase (GGT). Values are means, with their standard errors represented by vertical bars (*n* = 10). ^a,b^Mean values with different letters were considered to be significantly different (*P* < 0.05). CON: control; HMB: *β*-hydroxy-*β*-methylbutyrate; LPS: lipopolysaccharide.

As presented in Figure 2, the activities of serum ASAT and AKP were increased by LPS challenge, and the elevation of these parameters was nearly reversed to the level of control diet-fed pigs by HMB supplementation (*P* < 0.05). Serum activities of ALAT and GGT were not significantly different among groups (*P* > 0.05).

### 3.3. Liver mRNA Expression of Genes Related to the Energy Metabolism

**Figure 3:**
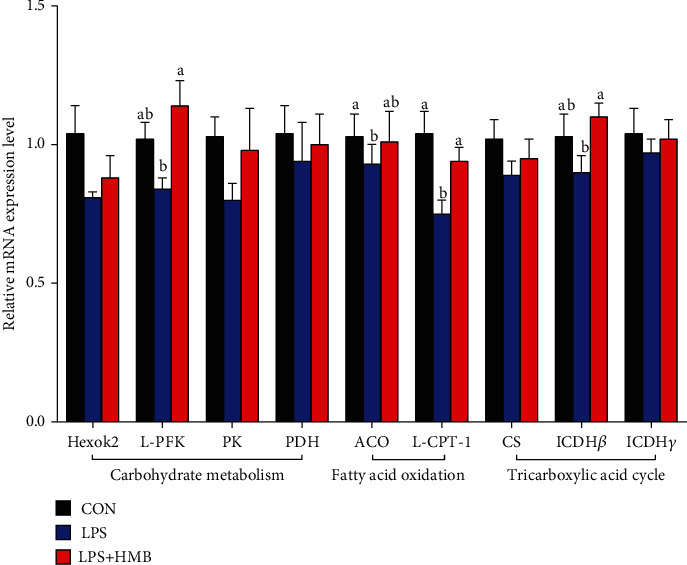
The mRNA expression of energy metabolism-related genes in the liver of piglets injected with LPS or saline. Carbohydrate metabolism-related genes: Hexok2: hexokinase 2; L-PFK: phosphofructokinase; PK: pyruvate kinase; PDH: pyruvate dehydrogenase. Fatty acid oxidation-related genes: ACO: acyl-coenzyme A oxidase; L-CPT-1: liver carnitine palmitoyltransferase I. Tricarboxylic acid cycle-related genes: CS: citrate synthase; isocitrate dehydrogenase *β*; ICDH *γ*: isocitrate dehydrogenase *γ*. Values are means, with their standard errors represented by vertical bars (*n* = 10). ^a,b^Mean values with different letters were considered to be significantly different (*P* < 0.05). CON: control; HMB: *β*-hydroxy-*β*-methylbutyrate; LPS: lipopolysaccharide.

The mRNA expression of genes involved in carbohydrate metabolism (hexokinase 2, Hexok2; phosphofructokinase, L-PFK; pyruvate kinase, PK; pyruvate dehydrogenase, PDH), fatty acid oxidation (acyl-coenzyme A oxidase, ACO; liver carnitine palmitoyltransferase I, L-CPT-1), and tricarboxylic acid cycle (citrate synthase, CS; isocitrate dehydrogenase *β*; isocitrate dehydrogenase *γ*, ICDH *γ*) in the liver of pigs was shown in Figure 3. Compared to the CON, LPS challenge significantly downregulated the mRNA expression of ACO and L-CPT-1 (*P* < 0.05) and had a tendency to downregulate the mRNA expression of L-PFK and ICDH *β* (*P* < 0.01). Among the LPS-challenged pigs, HMB supplementation upregulated the mRNA expression of L-PFK, L-CPT-1, and ICDH *β* (*P* < 0.05) and had a tendency to increase the ACO mRNA expression (*P* < 0.01).

### 3.4. Liver mRNA Expression of Genes Related to the Energy Sensing Network

**Figure 4:**
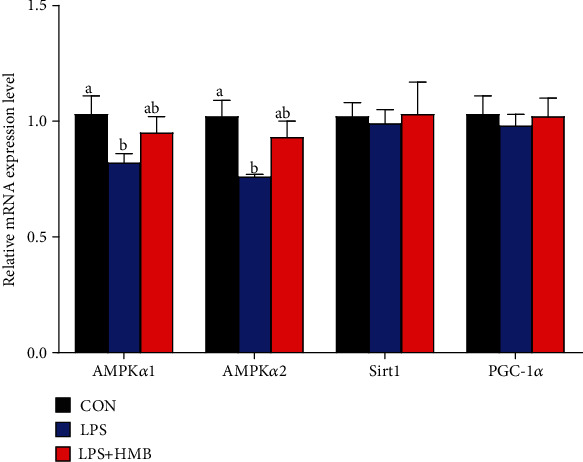
The mRNA expression of AMPK*α*1/2, Sirt1, and PGC-1*α* in the liver of piglets injected with LPS or saline. Values are means, with their standard errors represented by vertical bars (*n* = 10). ^a,b^Mean values with different letters were considered to be significantly different (*P* < 0.05). AMPK*α*1/2: AMP-activated protein kinase *α* 1/2; CON: control; HMB: *β*-hydroxy-*β*-methylbutyrate; LPS: lipopolysaccharide; PGC-1*α*: peroxisome proliferator-activated receptor-g coactivator-1*α*; SIRT1: silent information regulator transcript 1.

The mRNA expression of AMP-activated protein kinase *α* (AMPK*α*) 1/2, peroxisome proliferator-activated receptor *γ* coactivator 1*α* (PGC-1*α*), and silent information regulator 1 (SIRT1) in the liver of pigs was shown in Figure 4. Compared to the CON, significant decreases in the expression of AMPK*α*1 and AMPK*α*2 were shown in the liver of pigs after treatment with LPS (*P* < 0.05). Among the LPS-challenged pigs, HMB supplementation tended to increase the mRNA expression of AMPK*α*1 and AMPK*α*2 by 15.85% and 22.37%, respectively, but these decreases did not reach statistical difference (*P* > 0.05). Dietary treatments had no significant effects on the mRNA expression of Sirt1 and PGC-1*α* (*P* > 0.05).

### 3.5. Liver mRNA Expression of Proliferation Cell Nuclear Antigen (PCNA), Caspase-3, Tumor Necrosis Factor-*α* (TNF-*α*), and Heat Shock Protein 70 (HSP70)

**Figure 5:**
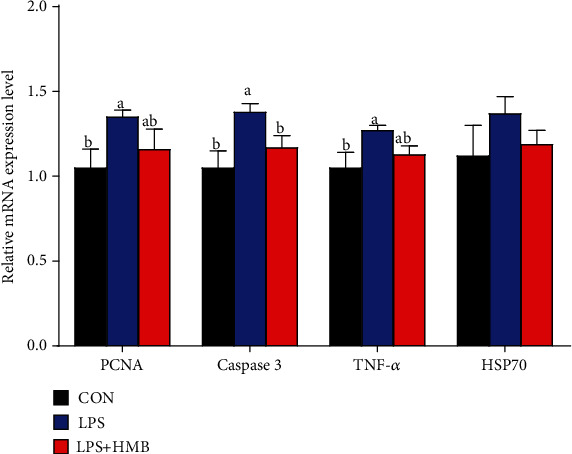
The mRNA expression of proliferation cell nuclear antigen (PCNA), caspase-3, tumor necrosis factor-*α* (TNF-*α*), and heat shock protein 70 (HSP70) in the liver of piglets injected with LPS or saline. Values are means, with their standard errors represented by vertical bars (*n* = 10). ^a,b^Mean values with different letters were considered to be significantly different (*P* < 0.05). CON: control; HMB: *β*-hydroxy-*β*-methylbutyrate; LPS: lipopolysaccharide.

As shown in Figure 5, compared to the CON, LPS challenge significantly upregulated the mRNA expression of liver PCNA, caspase 3, and TNF-*α* (*P* < 0.05). Compared to pigs treated with LPS, HMB supplementation significantly decreased the caspase 3 mRNA expression (*P* < 0.05) and downregulated the mRNA expression of PCNA and TNF-*α* by 14.07% and 11.02%, respectively, but this decrease did not reach statistical difference (*P* > 0.05). Dietary treatments did not significantly affect the mRNA expression of Sirt1 and PGC-1*α* (*P* > 0.05).

### 3.6. Liver mRNA Expression of TLR4 and Nucleotide-Binding Oligomerization Domain Protein (NODs) and Their Downstream Signals

**Figure 6:**
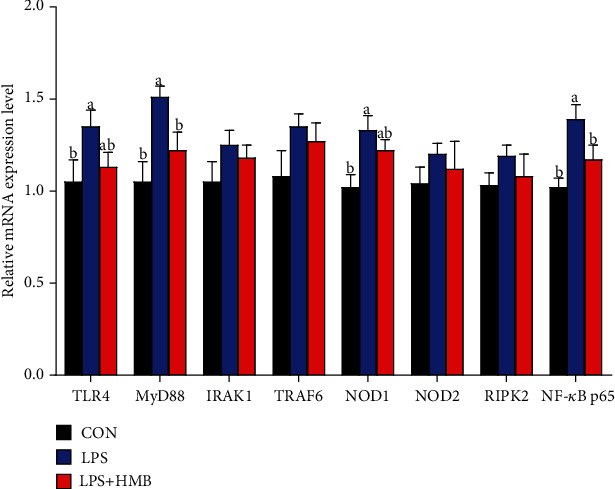
The mRNA expression of toll-like receptor 4 (TLR4) and nucleotide-binding oligomerization domain protein (NODs) and their downstream signaling molecules in the liver of piglets injected with LPS or saline. Values are means, with their standard errors represented by vertical bars (*n* = 10). ^a,b^Mean values with different letters were considered to be significantly different (*P* < 0.05). CON: control; HMB: *β*-hydroxy-*β*-methylbutyrate; IRAK1: IL-1 receptor-associated kinase 1; LPS: lipopolysaccharide; MyD88: myeloid differentiation factor 88; RIPK2: receptor-interacting serine/threonine-protein kinase 2; TRAF6: TNF-*α* receptor-associated factor 6.

As shown in Figure 6, LPS challenge significantly upregulated the mRNA expression of liver TLR4, MyD88, NOD1, and NF-*κ*B p65 relative to the CON (*P* < 0.05). Dietary HMB supplementation to the LPS-challenged pigs significantly downregulated the mRNA expression of MyD88 and NF-*κ*B p65 (*P* < 0.05) and decreased the mRNA expression of TLR4 and NOD1 by 16.30% and 8.27%, respectively, but this decrease did not reach statistical difference (*P* > 0.05).

### 3.7. Liver Protein Expression of P-AMPK*α* and Claudin-1

**Figure 7:**
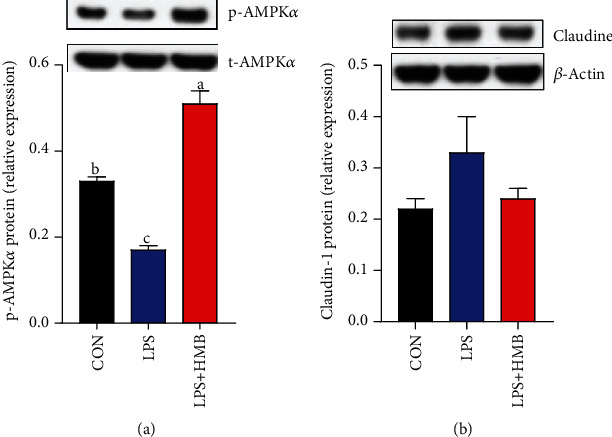
Western blot analysis of AMPK*α* phosphorylation (a) and claudin (b) expression in the liver of piglets injected with LPS or saline. Values are means, with their standard errors represented by vertical bars (*n* = 10). ^a,b,c^Mean values with different letters were considered to be significantly different (*P* < 0.05). CON: control; HMB: *β*-hydroxy-*β*-methylbutyrate; LPS: lipopolysaccharide.

As shown in Figure 7, no significant effect was observed for liver claudin-1 protein expression in response to dietary treatments (*P* > 0.05). The phosphorylation of AMPK*α* was downregulated by LPS challenge relative to the saline-injected pigs (*P* < 0.05). Compared to the LPS pigs, HMB supplementation significantly increased the protein expression of p-AMPK*α* (*P* < 0.05).

## 4. Discussion

The awareness of the roles of HMB in many biological and physiological processes including liver physiology is increasing [14, 15]. Our previous studies have revealed that dietary supplementation with 0.6% HMB mitigated growth suppression and intestinal injury of LPS-challenged weanling piglets [12, 13]. In the present study, we extended it into the liver to investigate the effects of 0.6% HMB supplementation on liver injury. We found that in response to LPS challenge, serum activities of ASAT and AKP (useful biochemical indicator of liver injury [8, 25]) were increased. These findings indicated that LPS could cause liver injury. Further evidence for a relationship between LPS challenge and liver injury comes from the findings of liver histopathological alterations, including infiltration of inflammatory leucocytes and karyolysis, karyopyknosis, vacuolation, and haemorrhage of hepatocytes. These results were consistent with other studies [2, 8]. Interestingly, HMB decreased serum activities of ASAT and AKP and ameliorated LPS-induced heptatocyte caryolysis, karyopycnosis, and fibroblast proliferation. These findings suggest that HMB offered a beneficial effect on the inhibition of liver injury.

To evaluate the mechanisms of HMB's mode of action, we initiated studies to analyze the mRNA expression of genes related to the energy metabolism. First, we found that HMB supplementation upregulated the mRNA expression of PFK, ACO, L-CPT-1, and ICDH *β* in the liver of LPS-challenged piglets. PFK is involved in catalyzing the first step of glycolysis, that is, the phosphorylation of glucose to glucose 6-phosphate to produce ATP and pyruvate [26]. ICDH *β*, a key enzyme in TCA cycle, transports metabolic intermediates from the cytosol into mitochondria to support the TCA cycle [27]. ACO and L-CPT-1 are implicated in the process of fatty acid oxidation, which is an important source of energy [7]. Therefore, the findings of the current study suggested that HMB, to some extent, could stimulate glycolysis, fatty acid oxidation, and TCA cycle to produce more ATP to mitigate LPS-induced energy stress in the liver of piglets. Such improved liver energy status prompted us to investigate whether HMB supplementation could activate AMPK signaling pathway in the liver of LPS-challenged piglets. AMPK is a cellular sensor of energy status; it functions to restore cellular ATP by switching off anabolic processes (further ATP consumption) in favor of catabolic processes (ATP generation). Enhanced AMPK activation has been reported to attenuate LPS-induced injury severity of tissues such as the liver, intestinal, and lung in animals [7, 13, 28]. In the present study, HMB supplementation upregulated the mRNA expression of AMPK*α*1/2 in the liver of LPS-injected piglets, accompanied by elevated AMPK*α* phosphorylation. Overall, these data suggested that HMB supplementation led to an improved energy status in the injured liver of LPS-injected piglets via activating AMPK signaling pathway.

Apart from energy, cell turnover including cell proliferation and apoptosis also affects tissue integrity and homeostasis [29, 30]. In particular, premature apoptosis occurs in response to errors in the signaling cascade of tissues under pathological condition. On the other hand, injured cells may proliferate uncontrolled and become a cancerous cell mass when they were unable to enter the apoptotic pathway [31]. In this study, we found that LPS challenge gave rise to hepatocyte proliferation and apoptosis, as evidenced by upregulated mRNA expression levels of PCNA and caspase-3. Other studies corroborate these results [32]. In agreement with expectations, the mRNA expression levels of PCNA and caspase-3 in the liver of LPS-challenged piglets were upregulated in response to HMB supplementation. These results revealed that inhibition of hepatocyte proliferation and apoptosis might be a contributing mechanism for the improved liver health in the LPS-injected piglets upon HMB supplementation.

Intrigued by decreased levels of serum proinflammatory cytokines upon HMB supplementation [12], we wondered whether HMB could ameliorate liver inflammatory response to improve liver integrity. Consistent with our serum TNF-*α* data, we found that hepatic TNF-*α* mRNA expression was upregulated following LPS challenge, and this elevation was reversed to the level of LPS-challenged piglets by HMB treatment. Emerging evidence has revealed that overproduction of proinflammatory cytokines leads to liver damage [33, 34]. Therefore, these data suggested that HMB might exert a role in reducing liver LPS-induced inflammation, which prompted us to perform in-depth studies to investigate the underlying mechanisms. TLR4 and NOD1/2 are the most popular signaling among others to respond primarily to LPS and to trigger the activation of inflammatory response [35, 36]. In this study, in good accordance with the alterations in serum TNF-*α* level and liver TNF-*α* mRNA expression, we observed that mRNA expressions of TLR4 and MyD88 (TLR4 signaling related genes), NOD1 (NOD signaling related genes), and NF-*κ*B p65 were increased in the liver of LPS-injected piglets, and then reversed by HMB. Therefore, inhibition of hepatic TLR4 and NOD signaling pathways in the LPS-injected piglets could be one possible mechanism to explain the ability of HMB to counter liver injury. Similar results were obtained in resistance-trained men (22.3 ± 2.4 years), in which HMB decreased circulating TNF-*α* and TNF-*α* receptor 1 expression during recovery [37]. Taken together, we expanded our scope from muscle and intestine to liver and demonstrated that HMB could also protect liver tissue from LPS-induced injury via inhibiting TLR4 and NOD signaling pathways.

In conclusion, our data demonstrate that dietary HMB supplementation could ameliorate liver injury in the LPS-challenged piglets. These beneficial effects of HMB might be associated with improved hepatic energy metabolism via regulating AMPK signaling pathway and reduced liver inflammation via modulating TLR4 and NOD signaling pathways. These findings contribute to developing new interventions to ameliorate liver injury and dysfunction in animals and humans with exposure to endotoxin.

## Figures and Tables

**Table 1:** Primers used for real-time quantitative PCR.

Genes	Forward (5′-3′)	Reverse (5′-3′)
HexoK 2	CTCATCACAACCGTTACCA	TGTCATTAGTGTCCTCATCC
L-PFK	CTGCACCGCATCATGGA	CCCCATCACCTCCAGAACA
PK	TCACTCCACAGACCTCAT	TACCTAGCCACCTGATGT
PDH	GCAGACTTACCGTTACCAT	GATAGCCGAGTTCTTCCAA
ACO	CTCGCAGACCCAGATGAAAT	TCCAAGCCTCGAAGATGAGT
L-CPT-1	GGACCGCCACCTGTTCTGCCTCTA	GCCCCCTCCGCTCGACACATAC
CS	TCTCAGCTCAGTGCAGCCATTACA	CTGCAACACAAGGTAGCTTTGCGA
ICDH *β*	TGTGGTTCCTGGTGAGAG	CGAGATTGAGATGCCGTAG
ICDH *γ*	GGTGGAGAGCCTCAAGAT	TGGTGGTGTTGTCTACGA
AMPK*α*1	AAATCGGCCACTACATCCTG	GGATGCCTGAAAAGCTTGAG
AMPK*α*2	AACATGGACGGGTTGAAGAG	CGCAGAAACTCACCATCTGA
Sirt1	CTGGAACAGGTTGCAGGAAT	CCTAGGACATCGAGGAACCA
PGC-1*α*	GATGTGTCGCCTTCTTGTTC	CATCCTTTGGGGTCTTTGAG
PCNA	TACGCTAAGGGCAGAAGATAATG	CTGAGATCTCGGCATATACGTG
Caspase-3	ACCCAAACTTTTCATAATTCA	ACCAGGTGCTGTAGAATATGC
TNF-*α*	TCCAATGGCAGAGTGGGTATG	AGCTGGTTGTCTTTCAGCTTCAC
COX2	ATGATCTACCCGCCTCACAC	AAAAGCAGCTCTGGGTCAAA
HSP70	GCCCTGAATCCGCAGAATA	TCCCCACGGTAGGAAACG
TLR4	TCAGTTCTCACCTTCCTCCTG	GTTCATTCCTCACCCAGTCTTC
MyD88	GATGGTAGCGGTTGTCTCTGAT	GATGCTGGGGAACTCTTTCTTC
IRAK1	CAAGGCAGGTCAGGTTTCGT	TTCGTGGGGCGTGTAGTGT
TRAF6	CAAGAGAATACCCAGTCGCACA	ATCCGAGACAAAGGGGAAGAA
NOD1	CTGTCGTCAACACCGATCCA	CCAGTTGGTGACGCAGCTT
NOD2	GAGCGCATCCTCTTAACTTTCG	ACGCTCGTGATCCGTGAAC
RIPK2	CAGTGTCCAGTAAATCGCAGTTG	CAGGCTTCCGTCATCTGGTT
NF-*κ*B p65	AGTACCCTGAGGCTATAACTCGC	TCCGCAATGGAGGAGAAGTC
*β*-Actin	TGCGGGACATCAAGGAGAAG	AGTTGAAGGTGGTCTCGTGG

ACO: acyl-coenzyme A oxidase; AMPK*α*: AMP-activated protein kinase *α*; CS: citrate synthase; Hexok 2: hexokinase2; HSP70: heat shock protein 70; ICDH *β*: isocitrate dehydrogenase *β*; ICDH *γ*: isocitrate dehydrogenase *γ*; IRAK1: IL-1 receptor-associated kinase 1; L-CPT-1: liver carnitine palmitoyltransferase I; L-PFK: 6-phosphofructokinase (liver type-like); MyD88: myeloid differentiation factor 88; NODs: nucleotide-binding oligomerization domain protein; PCNA: proliferation cell nuclear antigen; PDH: pyruvate dehydrogenase; PGC-1*α*: peroxisome proliferator-activated receptor-g coactivator-1*α*; PK: pyruvate kinase; RIPK2: receptor-interacting serine/threonine-protein kinase 2; SIRT1: silent information regulator transcript 1; TLR4: toll-like receptor 4; TNF-*α*: tumor necrosis factor-*α*; TRAF6: TNF-*α* receptor-associated factor 6.

## Data Availability

The data used to support the findings of this study are available from the corresponding author upon request.

## Supplementary Materials

Supplementary Materials Supplementary Table 1: composition and nutrient levels of the diets (air-dried basis, %).

## References

[1] Zhao D., Wu T., Yi D. (2017). Dietary supplementation with Lactobacillus casei alleviates lipopolysaccharide-induced liver injury in a porcine model. *International Journal of Molecular Sciences*.

[2] Kang P., Liu Y., Zhu H. (2018). The effect of dietary asparagine supplementation on energy metabolism in liver of weaning pigs when challenged with lipopolysaccharide. *Asian-Australasian Journal of Animal Sciences*.

[3] Chen J.-H., Yu G.-F., Jin S.-Y. (2015). Activation of *α*2 adrenoceptor attenuates lipopolysaccharide-induced hepatic injury. *International Journal of Clinical and Experimental Pathology*.

[4] Lai J. L., Liu Y. H., Liu C. (2017). Indirubin inhibits LPS-induced inflammation via TLR4 abrogation mediated by the NF-kB and MAPK signaling pathways. *Inflammation*.

[5] Zhang J., Xu X., Zhu H., Wang Y., Hou Y., Liu Y. (2019). Dietary fish oil supplementation alters liver gene expressions to protect against LPS-induced liver injury in weanling piglets. *Innate Immunity*.

[6] Wang L., Hou Y. Q., Yi D. (2015). Dietary supplementation with glutamate precursor *α*-ketoglutarate attenuates lipopolysaccharide-induced liver injury in young pigs. *Amino Acids*.

[7] Kang P., Liu Y., Zhu H. (2015). The effect of aspartate on the energy metabolism in the liver of weanling pigs challenged with lipopolysaccharide. *European Journal of Nutrition*.

[8] Yi D., Hou Y., Wang L. (2014). DietaryN-acetylcysteine supplementation alleviates liver injury in lipopolysaccharide-challenged piglets. *The British Journal of Nutrition*.

[9] Song Y. O., Kim M., Woo M. (2017). Chondroitin sulfate-rich extract of skate cartilage attenuates lipopolysaccharide-induced liver damage in mice. *Marine Drugs*.

[10] Duan Y., Li F., Li Y. (2016). The role of leucine and its metabolites in protein and energy metabolism. *Amino Acids*.

[11] Holecek M. (2017). Beta-hydroxy-beta-methylbutyrate supplementation and skeletal muscle in healthy and muscle-wasting conditions. *Journal of Cachexia, Sarcopenia and Muscle*.

[12] Duan Y., Zheng C., Zhong Y. (2019). Beta-hydroxy beta-methyl butyrate decreases muscle protein degradation via increased Akt/FoxO3a signaling and mitochondrial biogenesis in weanling piglets after lipopolysaccharide challenge. *Food & Function*.

[13] Zheng C., Song B., Duan Y. (2020). Dietary *β*-hydroxy-*β*-methylbutyrate improves intestinal function in weaned piglets after lipopolysaccharide challenge. *Nutrition*.

[14] Lattanzi B., Giusto M., Albanese C. (2019). The effect of 12 weeks of *β*-hydroxy-*β*-methyl-butyrate supplementation after liver transplantation: a pilot randomized controlled study. *Nutrients*.

[15] Sharawy M. H., El-Awady M. S., Megahed N., Gameil N. M. (2016). The ergogenic supplement *β*-hydroxy-*β*-methylbutyrate (HMB) attenuates insulin resistance through suppressing GLUT-2 in rat liver. *Canadian Journal of Physiology and Pharmacology*.

[16] Puiman P., Stoll B. (2008). Animal models to study neonatal nutrition in humans. *Current Opinion in Clinical Nutrition and Metabolic Care*.

[17] Merrifield C. A., Lewis M., Claus S. P. (2011). A metabolic system-wide characterisation of the pig: a model for human physiology. *Molecular BioSystems*.

[18] NRC (2012). *Nutrient Requirements of Swine (eleventh revised edition)*.

[19] Li Q., Liu Y., Che Z. (2012). Dietary L-arginine supplementation alleviates liver injury caused by Escherichia coli LPS in weaned pigs. *Innate Immunity*.

[20] Duan Y., Duan Y., Li F. (2016). Effects of supplementation with branched-chain amino acids to low-protein diets on expression of genes related to lipid metabolism in skeletal muscle of growing pigs. *Amino Acids*.

[21] Duan Y. H., Li F. N., Tan B. E. (2015). Myokine interleukin-15 expression profile is different in suckling and weaning piglets. *Animal Nutrition*.

[22] Li Y., Li F., Wu L. (2016). Effects of dietary protein restriction on muscle fiber characteristics and mTORC1 pathway in the skeletal muscle of growing-finishing pigs. *Journal of Animal Science and Biotechnology*.

[23] Duan Y., Li F., Li L., Fan J., Sun X., Yin Y. (2014). n-6:n-3 PUFA ratio is involved in regulating lipid metabolism and inflammation in pigs. *The British Journal of Nutrition*.

[24] Li F., Li Y., Tang Y. (2014). Protective effect of myokine IL-15 against H2O2-mediated oxidative stress in skeletal muscle cells. *Molecular Biology Reports*.

[25] Fernandez N. J., Kidney B. A. (2007). Alkaline phosphatase: beyond the liver. *Veterinary Clinical Pathology*.

[26] Currie P. D., Sullivan D. T. (1994). Structure and expression of the gene encoding phosphofructokinase (PFK) in Drosophila melanogaster.. *The Journal of Biological Chemistry*.

[27] MacDonald M. J., Brown L. J., Longacre M. J., Stoker S. W., Kendrick M. A., Hasan N. M. (2013). Knockdown of both mitochondrial isocitrate dehydrogenase enzymes in pancreatic beta cells inhibits insulin secretion. *Biochimica et Biophysica Acta (BBA) - General Subjects*.

[28] Zhao X., Zmijewski J. W., Lorne E. (2008). Activation of AMPK attenuates neutrophil proinflammatory activity and decreases the severity of acute lung injury. *American Journal of Physiology. Lung Cellular and Molecular Physiology*.

[29] Zhang X., Barile G., Chang S. (2009). Apoptosis and cell proliferation in proliferative retinal disorders: PCNA, Ki-67, caspase-3, and PARP expression. *Current Eye Research*.

[30] Sakao S., Taraseviciene-Stewart L., Lee J. D., Wood K., Cool C. D., Voelkel N. F. (2005). Initial apoptosis is followed by increased proliferation of apoptosis-resistant endothelial cells. *The FASEB Journal*.

[31] Schulte-Hermann R., Bursch W., Kraupp-Grasl B., Oberhammer F., Wagner A., Jirtle R. (1993). Cell proliferation and apoptosis in normal liver and preneoplastic foci. *Environmental Health Perspectives*.

[32] Leng W., Liu Y., Shi H. (2014). Aspartate alleviates liver injury and regulates mRNA expressions of TLR4 and NOD signaling-related genes in weaned pigs after lipopolysaccharide challenge. *The Journal of Nutritional Biochemistry*.

[33] Chen F., Liu Y., Zhu H. (2013). Fish oil attenuates liver injury caused by LPS in weaned pigs associated with inhibition of TLR4 and nucleotide-binding oligomerization domain protein signaling pathways. *Innate Immunity*.

[34] Kanuri G., Spruss A., Wagnerberger S., Bischoff S. C., Bergheim I. (2011). Role of tumor necrosis factor *α* (TNF*α*) in the onset of fructose-induced nonalcoholic fatty liver disease in mice. *The Journal of Nutritional Biochemistry*.

[35] Takeuchi O., Akira S. (2010). Pattern recognition receptors and inflammation. *Cell*.

[36] Moreira L. O., Zamboni D. S. (2012). NOD1 and NOD2 signaling in infection and inflammation. *Frontiers in Immunology*.

[37] Townsend J. R., Fragala M. S., Jajtner A. R. (2013). *β*-Hydroxy-*β*-methylbutyrate (HMB)-free acid attenuates circulating TNF-*α* and TNFR1 expression postresistance exercise. *Journal of Applied Physiology*.

