# Supplementary material for: Dietary Beta-Hydroxy Beta-Methyl Butyrate Supplementation Alleviates Liver Injury in Lipopolysaccharide-Challenged Piglets
Source: Oxid Med Cell Longev. 2021 Apr 1;2021:5546843. doi: 10.1155/2021/5546843 (PMC8035022; doi:10.1155/2021/5546843)
Supplement: Supplementary Materials — Supplementary Table 1: composition and nutrient levels of the diets (air-dried basis, %). [file 5546843.f1.pdf]

Supplementary Table 1. Composition and nutrient levels of the diets (air-dried basis, %)

Items	Basal diets	Basal diets + 0.60% HMB
Corn	30.14	29.44
Extruded corn	30.00	30.00
Soybean meal	9.00	9.14
Fish meal	7.00	7.00
Plasma protein powder	5.00	5.00
Whey powder	9.00	9.00
glucose	3.00	3.00
Soybean oil	3.80	3.98
HMB-Ca	0.00	0.60
Limestone	1.05	0.83
Choline chloride	0.10	0.10
Antioxidants	0.05	0.05
Citric acid	0.50	0.50
Salt	0.10	0.10
Vitamin premixa[a]	0.30	0.30
Mineral premixa[b]	0.15	0.15
Lys 98%	0.45	0.45
DL-Met	0.20	0.20
L-Thr	0.14	0.14
L-Trp	0.02	0.02
Total	100.00	100.00
Nutritional contents, %		
ME, MJ/kg	14.20	14.18
Crude protein	18.61	18.62
Ca	0.80	0.80
Total P	0.56	0.56
Available P	0.38	0.38
SID Lys	1.37	1.37
SID Methionine+Cysteine	0.75	0.75
SID Threonine	0.81	0.81
SID Tryptophan	0.22	0.22

[a]Vitamin premix supplied per kilogram of feed: 2200 IU Vitamin VA, 220 IU Vitamin D3, 0.5 mg Vitamin K3, 17.5 µg Vitamin B12, 3.5 mg riboflavin, 30 mg niacin, 10 mg d-pantothenic acid, 0.05 mg biotin, 0.3 mg folic acid, 1.0 mg thiamine, 7 mg pyridoxine, and 4.0 mg ethoxyquin.

[b]Mineral premix supplied per kilogram of feed: 150 mg Fe (FeSO4), 100 mg Zn ($ZnSO_4$), 30 mg Mn ($MnSO_4$), 25 mg Cu ($CuSO_4$), 0.5 mg I (KIO_3), 0.3 mg Co ($CoSO_4$), and 0.3 mg Se (Na_2SeO_3).
